# Electroresponse of weak polyelectrolyte brushes

**DOI:** 10.1140/epje/s10189-023-00341-3

**Published:** 2023-09-14

**Authors:** Christopher Balzer, Zhen-Gang Wang

**Affiliations:** Division of Chemistry and Chemical Engineering, California Institute of Technology, 1200 E California Blvd, Pasadena, CA 91125 USA

## Abstract

**Abstract:**

End-tethered polyelectrolytes are widely used to modify substrate properties, particularly for lubrication or wetting. External stimuli, such as pH, salt concentration, or an electric field, can induce profound structural responses in weak polyelectrolyte brushes, which can be utilized to further tune substrate properties. We study the structure and electroresponsiveness of weak polyacid brushes using an inhomogeneous theory that incorporates both electrostatic and chain connectivity correlations at the Debye–Hückel level. Our calculation shows that a weak polyacid brush swells under the application of a negative applied potential, in agreement with recent experimental observation. We rationalize this behavior using a scaling argument that accounts for the effect of the surface charge. We also show that the swelling behavior has a direct influence on the differential capacitance, which can be modulated by the solvent quality, pH, and salt concentration.

**Graphical Abstract:**

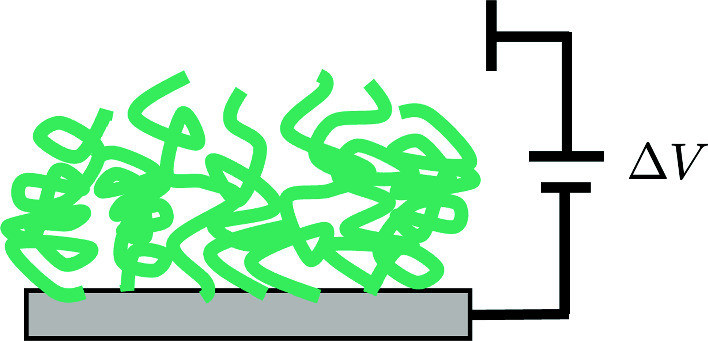

**Supplementary Information:**

The online version contains supplementary material available at 10.1140/epje/s10189-023-00341-3.

## Introduction

Grafting surfaces with polyelectrolytes is a promising route to functionalize surfaces for a wide variety of applications, including lubrication [1, 2], biosensing [3], anti-fouling [4–6], and assembly of nanoparticles [7, 8]. The conformation of a polyelectrolyte brush is closely tied to the charge state of the grafted chains [9, 10], which itself can be regulated by the local solution conditions [11–15], chemical details of the polymer sequence [16–18], and external forces (e.g. electric fields, confinement, etc.) [19, 20]. In this respect, surfaces grafted with weak polyelectrolytes are *smart* materials and the responsiveness to controllable variables enables surface properties to be readily tuned.

Adjusting the electrostatic condition of the surface is a facile and efficacious method to control conformation since voltage changes are simple in practice and the local electrostatic potential plays an important role in determining the local ionization [21–23]. Understanding the brush response to voltage changes is important in designing surfaces or materials involving weak polyelectrolyte brushes. The brush response to an electric field is coupled to all of the solution conditions and the possible conformations near the surface. Despite several theoretical studies on the electroresponsiveness of polyelectrolyte brushes [24–28], open questions remain about the electroresponsiveness of weak polyelectrolyte brushes [21]. For example, Senechal et al. recently observed two phenomena without clear explanation: (1) swelling of the brush under application of a negative (positive) electrostatic potential to a polyacid (polybase), and (2) strong hysteresis in the brush height upon cycling the electrostatic potential. The first phenomenon has been seen in other experimental studies [29, 30] and observed in the theoretical study by Okrugin et al.  [27], but the connection to other solution and surface properties, such as the pH and capacitance, is under-explored. The second phenomenon of hysteresis in weak polyelectrolyte brushes has been observed in other studies when varying the pH [31]. We expect the origin of the hysteresis to be similar in electroresponsive brushes. The open questions for each phenomenon call for a clear, mechanistic explanation in order to better design electroresponsive weak polyelectrolyte brushes.

In this work, we focus on the first question above: why does a polyacid brush swell when a negative potential is applied? To answer this question, we develop and apply an inhomogeneous thermodynamic theory to study the effect of the surface voltage on the swelling and de-swelling of a weak polyacid. The swelling of a polyacid upon applying a negative potential results from the free salt ions compensating the change in the surface charge density, leading to increases in the osmotic pressure. We extend the classical scaling approaches of polyelectrolytes to account for the effect of the surface charge density to rationalize this behavior. Finally, we explore implications of this mechanism by studying the capacitance of the weak polyacid brush, where the brush swelling is coupled to prominent peaks in the capacitance curves.

## Theoretical model

A model of weak polyelectrolyte brushes near a solid surface must take into account the local acid–base equilibria that drive ionization events along the polymer chain. For a polyelectrolyte with connected, titratable monomer units, proton binding and unbinding events among sites are correlated by their electrostatic and excluded volume interactions. These intrachain correlations are an essential aspect of polyelectrolyte ionization [18]. Aside from molecular simulation, which is computationally intensive, there is no widely accepted method to account for these correlations. The central difficulty is coupling the charge state of the polymer with its conformation, which often requires approximating or making assumptions of the underlying polymer chain structure [32]. For charge regulation, the random phase approximation [33], nearest-neighbor models [23, 34], and molecular dynamics sampling of conformations [22] have been developed to accomplish this. For inhomogeneous systems, a common approach is to use a perturbative, density-explicit free energy, such as is done in classical density functional theory [35, 36].

We proceed by developing a theoretical model that includes intrachain electrostatic correlations at the Debye–Hückel level. Treatment of electrostatics at the Debye–Hückel level is an approximation since it does not take into account the finite size of ions and is only rigorously applicable at low salt concentration [37]. We use this approximation since it results in simple analytical forms for the free energy and it captures the essential physics arising from electrostatic correlations, as we will demonstrate later. Such an approach is still an improvement over the mean-field theory, which has commonly been used to study weak polyelectrolytes.

Consider a polyelectrolyte solution made up of pH-responsive, linear polyelectrolytes, salt ions and water. Each monomer in the chain is a pH-responsive group whose charge state is determined by local acid–base equilibria. For simplicity, we consider each monomer to have only one dissociable proton (acid residue).

1 $$\begin{aligned} \begin{aligned} HM + H_2O \longleftrightarrow M^- + H_3O^+ \end{aligned} \end{aligned}$$

In the equations above, *M* is a generic acidic monomer. The reaction above is dictated by the acid–dissociation constant, $K_{a}$. Similarly, water can dissociate with $\textrm{pK}_a = 7$ for pure water at room temperature,

2 $$\begin{aligned} \begin{aligned} 2H_2O \longleftrightarrow OH^- + H_3O^+ \end{aligned} \end{aligned}$$

We consider the salt ions, denoted $+$ and − to be strong electrolytes with valency $z_+, z_-$ with $z_- < 0$. In the following development, we make several simplifications. We neglect the size of the bare proton and assume all the other species have the same size scale, denoted by *b*. This length scale also sets our volume scale for all species to be $v = b^3$. We take *v* to be the molecular volume of water so that $b = 3.1$ Å.

**Fig. 1:**
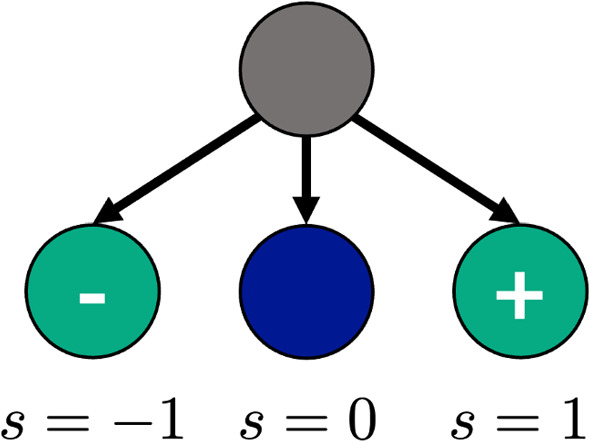
Schematic of Ising-like configurational states for monomers and water

From the acid–base equilibria, the monomers and water can each take on distinct states —neutral, protonated, or deprotonated. For an acid, only two states are available. To model the protonation and deprotonation, we introduce protonation variables, *s*, that denote the configurational state of a given species. For example, we use $s^w_i$ to denote the state of a given water molecule *i*. $s^w_i$ can take on values of −1, 0, or 1, corresponding to deprotonated, neutral, or protonated, respectively (Fig. 1). Likewise, the monomer state of the *j*-th monomer can be tracked the same way using $s^{M}_{j}$. Such a model is similar to that of Nakamura and Wang [38] in the context of salt-doped block copolymers and more recently, in classical density functional theory by Gallegos, Ong, and Wu [23].

The Helmholtz free energy is written as the sum of ideal and non-ideal contributions

3 $$\begin{aligned} \begin{aligned} F = F_{\mathrm{{id}}} + F_{\mathrm{{ev}}} + F_{\mathrm{{chm}}} + F_{\mathrm{{el}}} + F_{\mathrm{{DH}}} + F^{\mathrm{{ch}}}_{\mathrm{{DH}}} \end{aligned} \end{aligned}$$

where $F_{\mathrm{{ev}}}$ is a contribution from excluded volume (incompressibility), $F_{\mathrm{{chm}}}$ arises from acid–base reactions, $F_{\mathrm{{el}}}$ is the free energy for non-bonded electrostatic interactions, and the last two terms account for the electrostatic correlations at the Debye–Hückel level. The ideal part contains the mixing entropy from all of the species and states of each species,

4 $$\begin{aligned}{} & {} \beta F_{\mathrm{{id}}} = \int d{\textbf{r}} \sum _{\alpha = +,-} \left[ \rho _\alpha ({\textbf{r}}) \ln (\rho _\alpha ({\textbf{r}}) v_\alpha ) - \rho _\alpha ({\textbf{r}}) \right] \nonumber \\ {}{} & {} + \int d{\textbf{r}} \sum _{s_w} \left[ \rho _w({\textbf{r}},s_w) \ln (\rho _w({\textbf{r}},s_w) v_w) - \rho _w({\textbf{r}},s_w) \right] \nonumber \\ {}{} & {} + \int d{\textbf{R}} \sum _{\lbrace s_M \rbrace } \Big [ \rho _M({\textbf{R}},\lbrace s_M \rbrace ) \ln (\rho _M({\textbf{R}},\lbrace s_M \rbrace ) v_M^N) \nonumber \\ {}{} & {} \quad \quad \quad -\rho _M({\textbf{R}},\lbrace s_M \rbrace ) \Big ] \nonumber \\ {}{} & {} + \int d{\textbf{R}} \sum _{\lbrace s_M \rbrace } \rho _M({\textbf{R}},\lbrace s_M \rbrace ) \beta V_B({\textbf{R}},\lbrace s_M \rbrace ) \end{aligned}$$

where $\beta = 1/k_BT$ is the inverse temperature, $d{\textbf{R}}= \prod _{j=1}^N d{\textbf{r}}_{j}$, $\sum _{s_w}$ is the sum over the possible states of each water molecule, $\sum _{\lbrace s_M \rbrace }$ is the sum over the possible states of each monomer on each polymer chain, and $V_B({\textbf{R}},\lbrace s_M \rbrace )$ is the bonding potential for the polymer. The excluded volume interactions enter via the incompressibility constraint, creating a pressure-like field $\eta ({\textbf{r}})$ that acts as a Lagrange multiplier,

5 $$\begin{aligned} \begin{aligned} \beta F_{\mathrm{{ev}}} =&\int d{\textbf{r}} \beta \eta ({\textbf{r}}) \Big [ 1 - \sum _{\alpha = +,-} v_\alpha \rho _\alpha ({\textbf{r}}) \\&- \sum _{s_w} v_w \rho _w({\textbf{r}},s_w) \\&- \sum _{j=1}^N \sum _{s^M_{j}} v_M \rho ^M_{j}({\textbf{r}}, s^M_{j} )\Big ] \end{aligned} \end{aligned}$$

The chemical equilibria contributes

6 $$\begin{aligned} \begin{aligned} \beta F_{\mathrm{{chm}}}&= - \int d{\textbf{R}}\sum _{\lbrace s_M \rbrace } \rho _M({\textbf{R}},\lbrace s_M \rbrace ) \sum _{j=1}^N \beta \epsilon _M s^M_{j} \\ {}&+ \int d{\textbf{r}} \sum _{s_w} \rho _w({\textbf{r}},s_w) \beta \epsilon _w (s_w)^2 \\ {}&- \beta \lambda \Big [ \int d{\textbf{R}}\sum _{\lbrace s_M \rbrace } \rho _M({\textbf{R}},\lbrace s_M \rbrace ) \sum _{j=1}^N s^M_{j} \\ {}&+ \int d{\textbf{r}} \sum _{s_w} \rho _w({\textbf{r}},s_w) s_w \Big ] \end{aligned} \end{aligned}$$

where the first two terms account for the site binding energy and the last term introduces a Lagrange multiplier $\lambda$, which we call the *protonation potential* that is conjugate to the number of excess protons. In other words, one can use $\lambda$ to constrain the number of protons, enabling calculations with a constant pH reservoir. The value of $\lambda$ is determined by the pH as we show in the next section. The last term in the free energy is from direct Coulomb interactions,

7 $$\begin{aligned} \begin{aligned} \beta F_{\mathrm{{el}}}&= \int d{\textbf{r}} \Big [ \rho _c({\textbf{r}}) \beta e \psi ({\textbf{r}}) - \frac{1}{8\pi l_B} (\nabla \beta e \psi )^2 \Big ] \end{aligned}\end{aligned}$$

where $\rho _c({\textbf{r}}) = z_+ \rho _+({\textbf{r}}) + z_-\rho _-({\textbf{r}}) + \sum _{s_w} \rho _w({\textbf{r}},s_w) s_w + \sum _{j=1}^N \sum _{ s^M_j} \rho ^M_j({\textbf{r}}, s^M_j) s^M_j$ and $l_B=\beta e^2/4\pi \epsilon$ is the Bjerrum length, with $\epsilon$ being the dielectric permittivity. We assume a uniform dielectric constant of $l_B/b = 2.28$ corresponding to the dielectric constant of water at room temperature. We note that this assumption neglects the changing dielectric environment in the inhomogeneous system, which has shown to be important in the determining the charge state of weak polyelectrolytes [39].

The sum of the electrostatic correlations for disconnected ions and for chain connectivity can be written with a local approximation of the size-corrected Debye–Hückel free energy for ions of the same size,

8 $$\begin{aligned} \begin{aligned} F_{\mathrm{{DH}}} + F^{\mathrm{{ch}}}_{\mathrm{{DH}}} = \int d{\textbf{r}} f_{\mathrm{{DH}}}({\textbf{r}}) + \int d{\textbf{X}} f^{\mathrm{{ch}}}_{\mathrm{{DH}}} ({\textbf{X}}) \\ \end{aligned}\end{aligned}$$

where we lumped together the spatial and configurational (states) degrees of freedom into one variable ${\textbf{X}}$ ($\int d{\textbf{X}} = \int d{\textbf{R}} \sum _{\lbrace s_M \rbrace }$). For disconnected ions,

9 $$\begin{aligned} \begin{aligned} \beta f_{\mathrm{{DH}}} ({\textbf{r}}) =\frac{1}{4\pi d^3} \left[ \ln \big (1+ \kappa ({\textbf{r}}) d\big ) - \kappa ({\textbf{r}}) d + \frac{1}{2} \kappa ({\textbf{r}})^2 d^2 \right] \end{aligned}\nonumber \\\end{aligned}$$

where *d* is the Debye radius (we take $d=b$) and $\kappa ({\textbf{r}})$ is the local inverse Debye length, defined as

10 $$\begin{aligned} \begin{aligned} \kappa ({\textbf{r}})^2&= 4\pi l_B \Bigg [ z_+^2 \rho _+({\textbf{r}}) + z_-^2 \rho _-({\textbf{r}}) \\ {}&+ \sum _{s_w} \rho _w({\textbf{r}},s_w) (s_w )^2 \\ {}&+ \sum _{j=1}^N \sum _{ s^M_j} \rho ^M_j({\textbf{r}}, s^M_j) (s_j^M )^2 \Bigg ] \end{aligned}\end{aligned}$$

For the connectivity,

11 $$\begin{aligned} \begin{aligned} \beta f^{\mathrm{{ch}}}_{\mathrm{{DH}}} ({\textbf{X}}) = -\rho ({\textbf{X}}) \ln \Big ( g({\textbf{X}}) \Big ) \end{aligned}\end{aligned}$$

where $g({\textbf{X}})$ is the radial distribution function at contact. Using the TPT-1 approximation [40, 41] and the Debye-Hückel approximation for the correlation function [42] gives $g({\textbf{X}}) = \prod _{j=1}^{N-1} g({\textbf{x}}_j,{\textbf{x}}_{j+1}) = \prod _{j=1}^{N-1} \sqrt{ g({\textbf{r}}_j,s^M_j, s^M_{j+1} ) g({\textbf{r}}_{j+1},s^M_j, s^M_{j+1}) }$, where $g({\textbf{r}},s, s')= \exp \Big [ - \frac{s s' l_B /d }{ 1 + \kappa ({\textbf{r}}) d } \Big ]$. Defining $\zeta ({\textbf{r}},s, s') = \frac{s s' l_B /d}{2(1+ \kappa ({\textbf{r}})d)}$,

12 $$\begin{aligned} \begin{aligned}&\beta f^{ch}_{DH} ({\textbf{X}}) = \rho ({\textbf{X}}) \sum _{j=1}^{N-1} \big [\zeta ({\textbf{r}}_j,s^M_j, s^M_{j+1}) \\&\quad + \zeta ({\textbf{r}}_{j+1},s^M_j, s^M_{j+1})\big ] \end{aligned}\end{aligned}$$

The first variation of the grand potential energy $\Omega$ with respect to the electrostatic potential, the densities, and pressure field yields,

13 $$\begin{aligned}{} & {} \nabla ^2 \beta e \psi ({\textbf{r}}) = -4\pi l_B \rho _c({\textbf{r}}) \end{aligned}$$

14 $$\begin{aligned}{} & {} \begin{aligned}&\rho _\alpha ({\textbf{r}}) v_\alpha = \exp \Big [\beta \mu _\alpha - z_\alpha \beta e \psi ({\textbf{r}}) + v_\alpha \beta \eta ({\textbf{r}} )\\&\quad - \frac{\delta \beta f_{DH} }{\delta \rho _{\alpha }({\textbf{r}}) } - \frac{\delta \beta f^{ch}_{DH} }{\delta \rho _{\alpha }({\textbf{r}}) } \Big ] \end{aligned} \end{aligned}$$

15 $$\begin{aligned}{} & {} \begin{aligned}&\rho _w({\textbf{x}}) v_w = \exp \Big [\beta \mu _w - \beta \epsilon _w (s_w)^2 + \beta \lambda s_w - \beta e \psi ({\textbf{r}}) s_w \\ {}&\quad + v_w \beta \eta ({\textbf{r}}) - \frac{\delta \beta f_{DH} }{\delta \rho _{w}({\textbf{x}}) } - \frac{\delta \beta f^{ch}_{DH} }{\delta \rho _{w}({\textbf{x}}) } \Big ] \end{aligned} \end{aligned}$$

16 $$\begin{aligned}{} & {} \begin{aligned}&\rho _M({\textbf{X}}) v_M^N = \exp \Big [\beta \mu _M - \beta V_B({\textbf{X}}) + \sum _{j=1}^N \beta \eta ({\textbf{r}}) \\ {}&\quad + \sum _{j=1}^N \left[ \beta \epsilon _M + \beta \lambda - \beta e \psi ({\textbf{r}}) \right] s^M_j \\ {}&\quad - \frac{\delta \beta f_{DH} }{\delta \rho _{M}({\textbf{X}})} - \frac{\delta \beta f^{ch}_{DH} }{\delta \rho _{M}({\textbf{X}}) } \Big ] \end{aligned} \end{aligned}$$

17 $$\begin{aligned}{} & {} \begin{aligned}&\sum _{\alpha = +,-} v_\alpha \rho _\alpha ({\textbf{r}}) + \sum _{s_w} v_w \rho _w({\textbf{r}},s_w) \\ {}&\quad + \sum _{j=1}^N \sum _{s^M_{j}} v_M \rho ^M_{j}({\textbf{r}}, s^M_{j} ) = 1 \end{aligned} \end{aligned}$$

where the local potentials for the electrostatic correlation terms are given in the Supplementary Information. Note that each element in the sum over $\eta ({\textbf{r}})$ in Eq. 16 is identical, yielding *N* total factors of $\eta ({\textbf{r}})$. Substituting a normalized Gaussian potential for the bonding potential, the monomer state density for internal monomers is

18 $$\begin{aligned} \begin{aligned} \rho ^M_j&({\textbf{r}}, s^M_{j-1},s^M_j,s^M_{j+1}) v_M = \int d\mathbf {X'} \rho _M(\mathbf {X'}) v_M \times \\ {}&\delta ({\textbf{r}} - \mathbf {r'}_j) \delta _{s^M_{j-1},s^{M'}_{j-1}} \delta _{s^M_j,s^{M'}_j} \delta _{s^M_{j+1},s^{M'}_{j+1}} \\ = \,&e^{\beta \mu _M} e^{-\beta \omega ({\textbf{r}},s^M_{j-1},s^M_j,s^M_{j+1})} \times \\ {}&q_j({\textbf{r}},s^M_{j-1},s^M_j,s^M_{j+1}) q_j^*({\textbf{r}},s^M_{j-1},s^M_j,s^M_{j+1}) \end{aligned}\end{aligned}$$

where the effective field $\omega$ generally depends on the position ${\textbf{r}}$ and monomer state $s_j$ as well as adjacent states, $s_{j-1}$ and $s_{j+1}$. Similarly, on the chain ends, we have

19 $$\begin{aligned} \begin{aligned} \rho ^M_1&({\textbf{r}},s^M_1,s^M_{2}) v_M = e^{\beta \mu _M} e^{-\beta \omega ({\textbf{r}},s^M_1,s^M_{2})} \times \\ {}&q_1({\textbf{r}},s^M_1,s^M_{2}) q_1^*({\textbf{r}},s^M_1,s^M_{2}) \end{aligned} \end{aligned}$$

20 $$\begin{aligned} \begin{aligned} \rho ^M_N&({\textbf{r}},s^M_{N-1},s^M_{N}) v_M = e^{\beta \mu _M} e^{-\beta \omega ({\textbf{r}},s^M_{N-1},s^M_{N})} \times \\ {}&q_N({\textbf{r}},s^M_{N-1},s^M_{N}) q_N^*({\textbf{r}},s^M_{N-1},s^M_{N}) \end{aligned}\end{aligned}$$

The propagators *q* and $q^*$ can be defined recursively. The explicit expressions for the effective fields and propagators can be found in the Supplementary Information.

One can obtain the total density for a given monomer by summing over the configurational variables

21 $$\begin{aligned} \begin{aligned} \rho ^M_j({\textbf{r}}) = \sum _{s^M_{j-1}} \sum _{s^M_{j}} \sum _{s^M_{j+1}} \rho ^M_j({\textbf{r}},s^M_{j-1},s^M_j,s^M_{j+1}) \end{aligned}\end{aligned}$$

For a polymer brush, the first monomer is constrained to the surface so that the initial propagator is given by

22 $$\begin{aligned} \begin{aligned} q_1(z,s^M_{1},s^M_{2}) = e^{\beta \omega (z,s^M_{1},s^M_{2})} \delta (z) \end{aligned}\end{aligned}$$

when the variation is only in the z-direction (dimensionless *z*). Likewise, we have the condition related to the grafting density

23 $$\begin{aligned} \begin{aligned} \sigma _g&= \int dx \rho ^M_1(x) \\ {}&= \frac{e^{\beta \mu _M}}{v_M/b} \sum _{s^M_1} \sum _{s^M_2} q_1^*(0,s^M_{1},s^M_{2}) \end{aligned}\end{aligned}$$

which allows the chemical potential of the polyelectrolyte chain to be determined from the constraint of the grafting density. The fraction of any state for each species can be determined from the density expressions. For example, the amount of $H_3O^+$ can be determined from the states of water,

24 $$\begin{aligned} \begin{aligned} f_{H_3O^+} ({\textbf{r}}) = \frac{\rho _w({\textbf{r}},+1) }{\sum _{s_w = -1,0,+1} \rho _w({\textbf{r}},s_w) } \end{aligned}\end{aligned}$$

### Determining binding constants

So far, the binding constants $\epsilon _w$ and $\epsilon _M$ and the protonation potential $\lambda$ have not been specified. For convenience, we can define the following constants $K^w = \exp (\beta \epsilon _w)$ and $K^M = \exp (\beta \epsilon _M)$. To obtain values for $K^w$ and $K^M$ for the acid–base equilibria, we connect the expressions to the conventional notation. To do this, we use the fact that for pure water the equilibrium constant is $K_a^w \approx 10^{-14} \ \textrm{M}^2$ and the total water density is $c^0_{w} \approx 55.5 \ \textrm{M}$.

25 $$\begin{aligned} \begin{aligned} \frac{[H_3O^+] [OH^-] }{ [H_2O]^2 } = \frac{10^{-14} }{ [H_2O]^2 } = (K^w)^2 \end{aligned}\end{aligned}$$

where the concentrations are determined by the fractions and total density, $[H_3O^+]= c^0_w f_{H_3O^+}$. Since the fraction of $f_{H_2O} \approx 1$, then $K^w = 10^{-8.744}$. Similarly for the equilibria for each monomer in the absence of an electric field and in a dilute solution,

26 $$\begin{aligned} \begin{aligned} \frac{ [H_3O^+] [M^-] }{ [H_2O][HM] } = K^w K^{M} = K_{a,0} / c^0_{w} \end{aligned}\end{aligned}$$

where $$ K_{a,0}$$ is the acid dissociation constant for the monomers so that $$K^{M} = 10^{7-pK_{a,0}}$$. By specifying the $$pK_{a,0}$$, the dissociation constant can be obtained.

The protonation potential is determined by the pH and the total water concentration in the absence of an electric field. Starting with the definition of Eq. 24 and using the density for pure water from Eq. 15,

27 $$\begin{aligned} \begin{aligned} f_{H_3O^+} = \frac{K^w e^{\beta \lambda } }{ 1+ K^w e^{\beta \lambda } + K^w e^{-\beta \lambda } } \end{aligned}\end{aligned}$$

Substituting $$ f_{H_3O^+} = [H_3O^+]/c^0_w$$ and using the fact that $$[H_3O^+] = 10^{-pH}$$ in pure water,

28 $$\begin{aligned} e^{\beta \lambda } {=} 10^{-pH} \frac{1 {+} \sqrt{1 {+} 4 c_w^0 (K^w)^2 \times 10^{pH} (1{-} 10^{-pH }/c_w^0) } }{ 2 c_w^0 K^w (1 {-} 10^{-pH }/c_w^0) }\nonumber \\ \end{aligned}$$

## Results and discussion

### Bulk titration

**Fig. 2:**
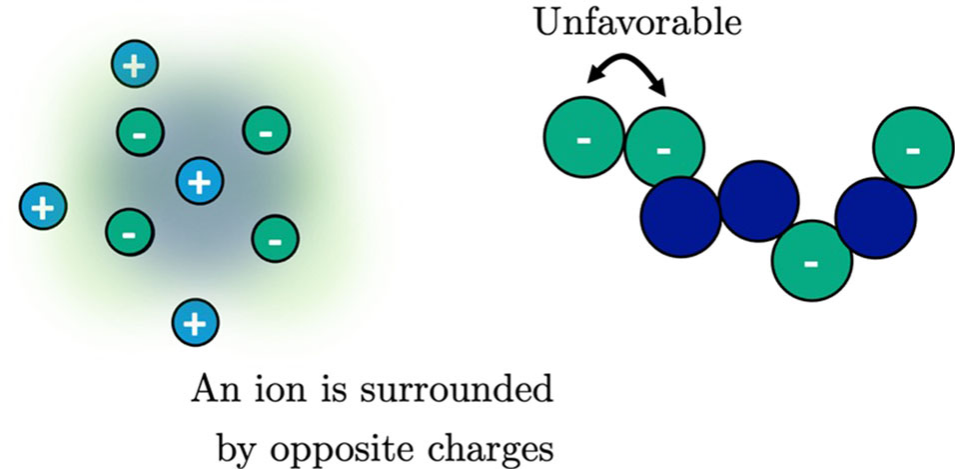
Schematic showing the effect of Debye-Hückel correlations. (left) Like-charged ions are screened due to the local structuring from oppositely-charged ions. (right) Ionization of two adjacent monomers is unfavorable due to the intrachain repulsion

We begin by considering the bulk titration in a polyelectrolyte solution. In the absence of electrostatic correlation, the charge state of the monomers in a uniform bulk solution only depends on the pH and the pK$_a$. Electrostatic correlations render the charge state dependent on properties such as the degree of polymerization and local salt concentration (Fig. 2). Adding in the Debye–Hückel correlations for disconnected ions favors ionization of the monomers due to the local screening environment. Like-charged ions have less repulsion with each other arising from the ion structuring around each ion. For a connected chain, the correlations at the nearest-neighbor level suppress ionization since there is a penalty for two adjacent monomers to be ionized.

**Fig. 3:**
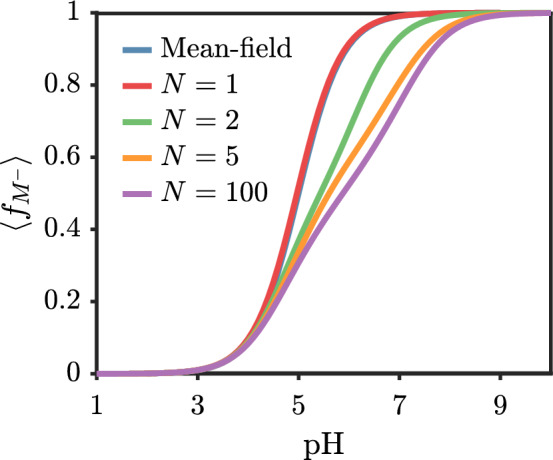
Average fraction of ionized monomers on polyacid versus solution pH for various chain lengths with $pK_{a,0}=5.0$ and the added salt $c_\pm = 10 \ \textrm{mM}$. The mean-field result (blue) does not depend on the degree of polymerization and nearly overlaps with the $N=1$ curve with correlations. The rest of the curves include Debye–Hückel level correlations

The nearest-neighbor (TPT-1) level approach to the electrostatic contribution to the chain connectivity correlations creates a weak dependence of the charge state on the chain length. TPT-1 level treatment of electrostatic correlation is known to underestimate the electrostatic correlation and be relatively insensitive to chain length [32]. The nearest-neighbor nature of TPT-1 here makes it similar to the transfer matrix theory developed by Sing and coworkers [34]. Figure 3 shows the bulk titration behavor for a dilute acid solution for different chain lengths. As expected, increasing the chain length decreases the ionized fraction due to the penalty of ionizing adjacent monomers. The effect saturates rather quickly with $N=5$ and $N=100$ being quite similar. The largest disparities from the mean-field result (no correlation, $F_{\mathrm{{DH}}} + F^{\mathrm{{ch}}}_{\mathrm{{DH}}} = 0$) occur for pH values above the pK$_a$ since the polymer is more likely to be ionized at those conditions. Additionally, because we track the charge fraction of each monomer, we are able to capture some sequence-dependent phenomena such as the small shoulder peak that appears near pK$_a=5$ for $N=100$. This feature appears due to the driving force of ionization forcing ionization of neighboring monomers, which, in the limit of strong neighbor–neighbor repulsion, appears as a plateau in the titration curve [18, 43].

**Fig. 4:**
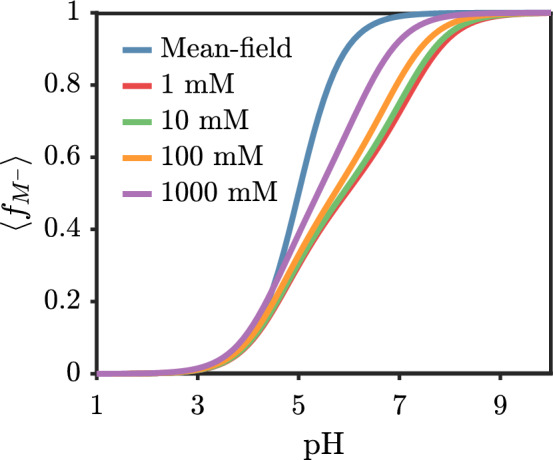
Average fraction of ionized monomers on polyacid versus solution pH for various added salt concentrations with $pK_{a,0}=5.0$ and chain length $N = 100$. The mean-field result (blue) does not depend on the salt concentration. The rest of the curves include Debye–Hückel level correlations

For a fixed chain length, adding salt increases the amount of screening and decreases the penalty for adjacent ionized monomers. Figure 4 shows that increasing the salt concentration increases the degree of ionization. Even for a 1 M solution, there is still a noticeable difference between the mean-field result and that with correlations. These differences will undoubtedly play a role in the brush system, where the strong inhomogeneity induced by the surface creates widely varying local conditions for the weak polyelectrolyte.

**Fig. 5:**
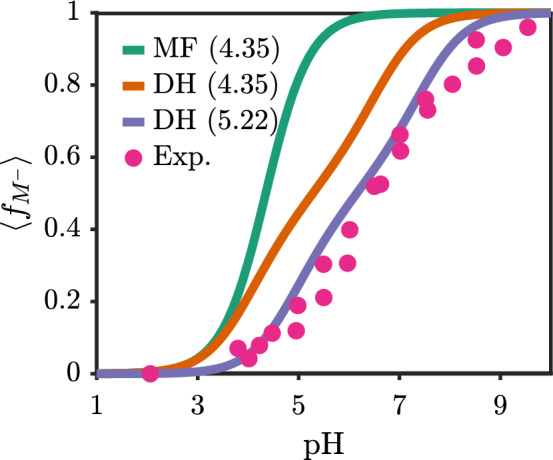
Comparison of bulk titration behavior across mean-field, Debye–Hückel correlations, and experiment. Experimental values are for poly(acrylic acid) are combined from Refs. [44, 45]. For calculations, an added salt concentration of 1 mM, acid monomer concentration of 1.35 mM, and $N=100$ are used. Numerical values in legend indicate value of $pK_{a,0}$. MF = Mean-field, DH = Debye–Hückel correlations, Exp=Experiment

We briefly offer a comparison to experiments in Fig. 5. The experimental value for the pK$_a$ of the monomeric form of poly(acrylic acid) is 4.35 from Ref. [33]. Using this value for pK$_{a,0}$, the mean-field result fails to capture the correct ionization behavior, leading to an overestimate due to the lack of connectivity correlations. The result with correlations yields the correct slope of the titration curve but appears shifted. A detailed classical density functional theory based on the mean spherical approximation (MSA) and nearest-neighbor chain connectivity correlation predicted the thermodynamic pK$_a$ to be 5.22 for poly(acrylic acid) [18]. Using this value leads the curve with correlations (purple curve) to nearly overlap with the experimental data. For this reason, we are optimistic that even at the Debye–Hückel, we are able to capture the salient physics of ionization in weak polyelectrolytes.

### Brush structure at neutral surface

**Fig. 6:**
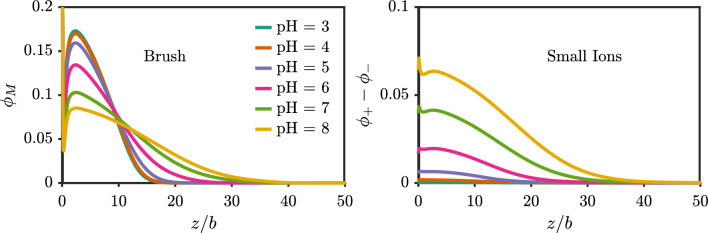
Density profiles near a neutral surface for different values of pH with $pK_{a,0}=5$. (left) Polyacid brush. (right) Net charge density from small ions. The salt concentration is 10 mM, the chain length is $N=50$ and the grafting density is $\sigma _g b^2 = 0.03$

The structure of a polyelectrolyte brush is closely tied to its charge state [46]. A highly charged polyelectrolyte grafted to a surface will tend to extend away from the surface to minimize the ion–ion repulsion with other grafted chains. The chains cannot stretch arbitrarily far owing to the entropic cost of chain stretching—available chain conformations are reduced in the extended state. The balance between these two effects gives rise to the equilibrium brush height [9]. Figure 6 shows a well-known but important result of the brush profile on a charge-neutral surface for different values of the pH. For a polyacid, as the pH increases, the average ionization in the brush increases, similar to the bulk titration behavior. As shown in Fig. 6, the brush takes on a more extended conformation at a pH of 8 versus that of 3. Counterions from the salt solution enter the brush region to compensate the charge from the brush. Figure 6 also shows that the length scale of the net charge from the small ions extends as far as the brush profile. Figure S1 shows the corresponding spatially dependent brush ionization and local concentration of hydronium, indicating lower overall ionization closer to the surface.

The average fraction of ionized monomers in the brush can be calculated using

29 $$\begin{aligned} \begin{aligned} \langle f_{M^-} \rangle = \frac{\int dz \rho _M(z,-1)}{\int dz \sum _{s_M}\rho _M(z,s_M)} \end{aligned}\end{aligned}$$

**Fig. 7:**
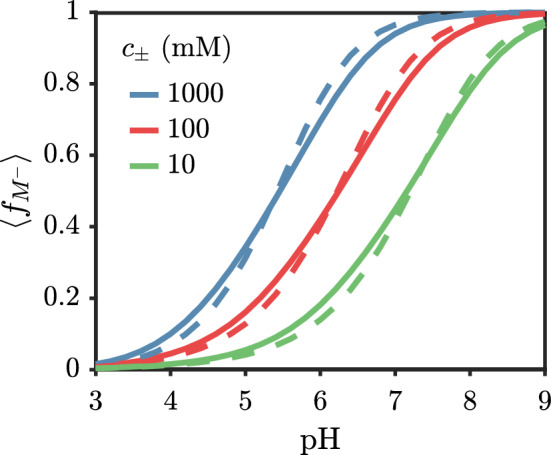
Average fraction of ionized monomers for a polyacid brush grafted to a neutral surface versus the solution pH with $pK_{a,0}=5$. (solid) Theory with Debye–Hückel correlations. (dashed) Mean-field theory (no correlations). The chain length is $N=50$ and the grafting density is $\sigma _g b^2 = 0.03$

Figure 7 indicates that in both the mean-field and theory with correlations, the average ionized fraction can span the full range from 0 to almost 1 by changing the pH from 3 to 9. What is somewhat surprising is how similar the mean-field result is to the theory with correlations, despite their marked differences in the bulk titration behavior. This similarity is a consequence of the strong inhomogeneity in the local electrostatic potential [23]. The local electrostatic potential is a one-body term on each monomer that directly influences the charge state and plays a relatively larger role than the net effect of correlations near a surface compared to in a bulk solution.

**Fig. 8:**
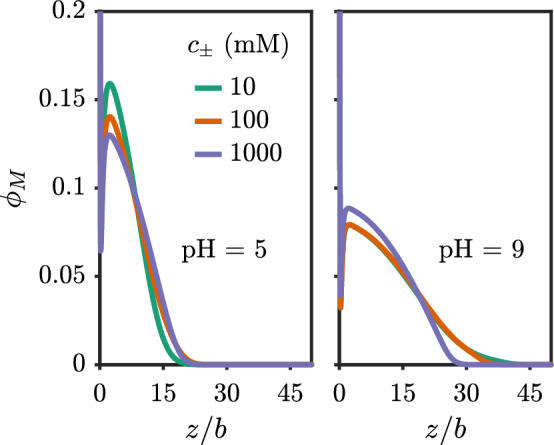
Polyacid brush density profile near a neutral surface for different salt concentrations at (left) pH = 5 and (right) pH = 9 with $pK_{a,0}=5$. The chain length is $N=50$ and the grafting density is $\sigma _g b^2 = 0.03$

At a fixed pH, there are competing effects for the brush height upon the addition of salt. On one hand, the addition of salt screens the ion–ion repulsion, which generally reduces the penalty for ionization, leading to a higher ionized fraction with increasing salt. This tends to extend the brush. On the other hand, screening ion–ion repulsion reduces the penalty of contracting the brush. Figure 8 shows the effect of adding salt at pH=5 and pH=9. For pH=5, adding salt increases the brush height, while adding salt decreases the brush height for pH=9. This indicates that the brush is in-between the *osmotic* and *salted* regimes depending on the pH [47]. The onset of the salted brush regime is known to occur in the range of 10 mM for fully charged polyelectrolytes [31].

### Response to surface potential

**Fig. 9:**
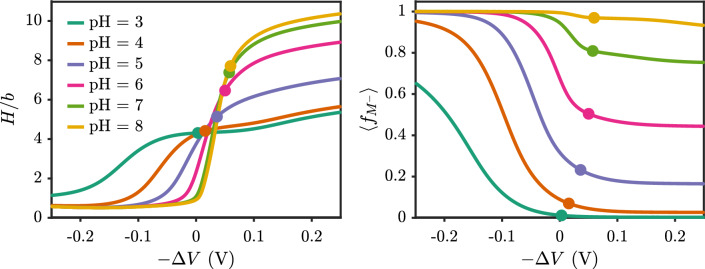
Effect of applied potential on the brush conformation and charge for $pK_{a,0}=5$. (left) Brush height and (right) average ionized fraction as a function of the negative of the applied electrostatic potential on the surface. Filled circles correspond to conditions of a neutral surface. The salt concentration is 100 mM, the chain length is $N=20$, and the grafting density is $\sigma _g b^2 = 0.01$

In the last section, we considered polyacid brushes grafted to neutral surfaces. For the electroresponsive behavior, the guiding parameter is the electrostatic potential at the surface. When the surface is neutral, a grafted polyacid carries negative charge and will induce a negative surface potential. The point of zero potential occurs when the surface carries a positive surface charge. Figure 9 shows the effect of the surface potential on the brush height. The abscissa in the plot is given as the negative value of the applied potential since that is the language of the motivating experimental work [21]. Figure S2 shows a progression of brush profiles upon applying a potential. There are a few clear trends from these plots. For any given pH: (1) a more negative applied potential swells the polyacid brush and (2) there is a range of applied potential where the brush height changes rapidly (in the range of $\Delta V$ from 0 to 0.1 V for pH=8). At the same time, the average ionized fraction in the brush monotonically decreases for negative potentials. How can the brush swell if the ionized fraction decreases?

To answer this question, we turn to classical scaling arguments of polyelectrolyte brushes [7, 48] and use similar reasoning as Borisov and coworkers [27, 49], who considered strong and weak polyelectrolyte brushes near charged surfaces. Viewing the problem through a brush-centric lens does not account for the effect of the surface condition. When a negative potential is applied to a surface, the surface charge density must decrease since the capacitance is positive. When the surface charge density decreases, the polyacid will either be less attracted to a positively charged surface or will be more repelled from a negatively charged surface. In the osmotic and salted brush regime, the equilibrium brush height is determined by the balance of osmotic pressure from small ions in the brush region and the stretching of the brush. Dividing the system into a bulk solution in contact with a brush region, the difference in the osmotic pressure between the bulk solution and the brush region is $f_{osmo} \sim \frac{f \phi }{\sigma _g b^3}$ (osmotic brush) and $f_{osmo} \sim \frac{f^2 \phi _{\text {brush}}^2 }{\sigma _g b^6 C_s}$ (salted brush) and the elastic force for a polyelectrolyte is $f_{el} \sim H/Nb^2 \sim \sigma _g b /\phi _{\text {brush}}$, where $\phi _{\text {brush}} = N b^3 \sigma _g / H$ is the average monomer volume fraction in the brush region  [9, 14]. The balance of these two forces leads to the classic scaling relation for the brush height, $H \sim N b f^{1/2}$ (osmotic) and $H \sim N b^{2/3} \sigma _g^{1/3}C_s^{-1/3} f^{2/3}$ (salted).

From these relationships, applying a negative potential decreases the average ionization *f* and the brush should contract. To add the contribution from the surface charge, we treat it similarly to the fixed, immobilized charge of the monomers in the brush region so that the ions in the bulk solution *see* a combined charge of $Q_s/H + \phi _{\text {brush}} f$ in the brush region. For salt-free polyelectrolyte brushes, it has previously been shown that the overall immobilized charge (brush and surface charge contributions) is the main quantity rather than the distribution between the surface and brush [49]. For the salted brush, the ion-ion repulsion is then $f_{osmo} \sim \frac{f^2 \phi _{\text {brush}}^2 \left( 1 - \frac{Q_s}{Nf\sigma _g }\right) ^2}{\sigma _g b^6 C_s}$ for a polyacid. The brush height scaling becomes $H \sim N b^{2/3} \sigma _g^{1/3}C_s^{-1/3} \left( f - \frac{Q_s}{N\sigma _g}\right) ^{2/3}$. For the osmotic brush, $H \sim N b \left( f - \frac{Q_s}{N\sigma _g}\right) ^{1/2}$. The scaling analysis above is not applicable in the regime where the polyelectrolyte is fully collapsed on the surface. In that case, the relevant force balance is the attraction of the brush to the surface and the force required to confine the brush to a thin surface layer [50].

**Fig. 10:**
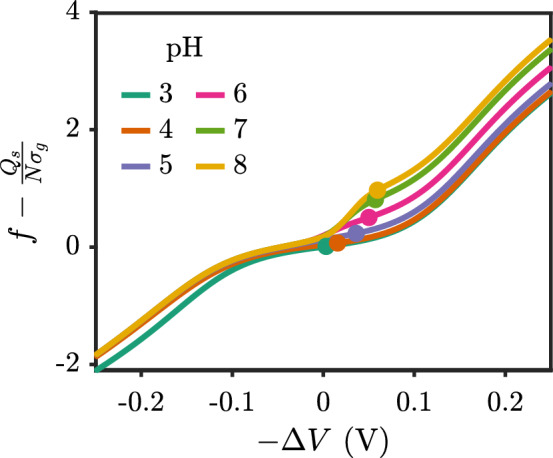
Overall charge fraction as a function of the negative of the applied electrostatic potential on the surface with $pK_{a,0}=5$. Filled circles correspond to conditions of a neutral surface. The salt concentration is 100 mM, the chain length is $N=20$ and the grafting density is $\sigma _g b^2 = 0.01$

The scaling analysis above indicates that the brush height is determined by the overall charge fraction, $f - \frac{Q_s}{N\sigma _g}$. For a *fixed* charge fraction of a polyelectrolyte, the overall charge fraction is monotonic with the surface charge density. For weak polyelectrolytes, the charge fraction *f* decreases as $-Q_s$ increases, leading to a competition between change in ionization and the change in surface charge density. When applying a negative potential to the surface, the energy can either go toward neutralizing monomers or confining more small ions in the brush region. One can also work out an approximate condition on the differential capacitance from the scaling relationship, where brush extension upon applying a negative potential will occur when $C_d > N\sigma _g \frac{\partial f}{\partial \Delta V}$. The dependence of *f* on the surface potential is not obvious, but can be calculated numerically. Figure 10 shows that the overall charge fraction for the polyacid brush considered here is monotonically increasing with the negative applied potential, indicating that the brush should swell. The plot shows data for 100 mM, and while not shown, the same trend is true for salt concentrations of 10 mM and 1000 mM. We do not expect the scaling exponents to be accurate for this system because we are considering short chains and the correlations (i.e. electrostatic and excluded volume) adjust the expression for the ion-ion repulsion. Other polyelectrolyte brush systems also have shown scaling that deviates from the predicted scaling, even at the mean-field level [51].

**Fig. 11:**
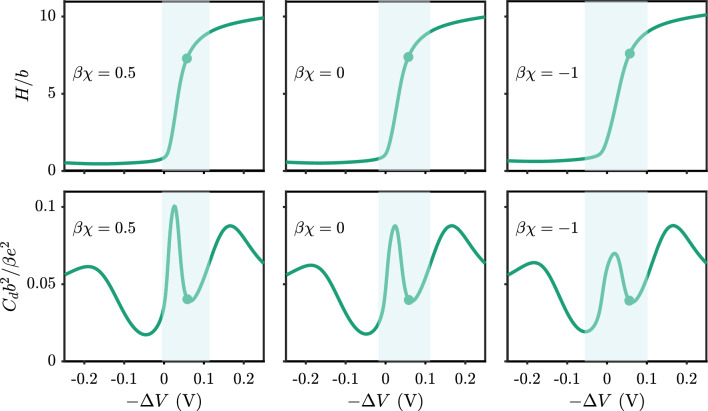
Brush extension (top row) and differential capacitance (bottom row) as a function of the negative of the applied electrostatic potential for different solvent qualities for $pK_{a,0}=5$. The solvent quality includes $\beta \chi = 0.5$ (left), $\beta \chi = 0$ (middle) and $\beta \chi = -1$ (right). Filled circles correspond to conditions of a neutral surface. The pH is 7, the salt concentration is 100 mM, the chain length is $N=20$ and the grafting density is $\sigma _g b^2 = 0.01$

Having rationalized the swelling behavior from electrostatic arguments, we investigate some implications of the electroresponsiveness. We will analyze the differential capacitance of the polyacid brush in different solvent and solution conditions since the capacitance of weak polyelectrolyte brushes has not been widely studied. Most existing work by Szleifer and co-workers focused on a limited range of voltages [52, 53] such that many of the features in the capacitance curves we report were not apparent. Figure 11 shows the brush height when applying a surface potential and the corresponding capacitance for different solvent conditions, $\chi$-type interactions. The Supplementary Information shows how we incorporate the solvent quality into our theory. As shown in each column of the figure, the region where the brush height rapidly changes corresponds to a peak in the differential capacitance. As the brush expands, counterions rush into the brush region to compensate the surface charge, leading to an increase in the charge stored in the EDL. For the polyacid brush, the brush height response to the potential is closely connected to the capacitive performance. Moving from left to right, the panels show the same plots for the brush in increasingly better solvent quality. Increasing the solvent quality increases the propensity of the brush to extend, whereby the brush can resist electrostatic forces that tend to collapse the brush on the surface. The net result is that the brush height changes more slowly with increasing solvent quality. For the capacitance, less rapid changes in the brush height leads to less prominent peaks in the capacitance. So for capacitance applications, the optimal conditions are to have a poor enough solvent to cause rapid extension upon charging without having too poor of a solvent, where the electrostatic forces cannot overcome the barrier to extend the brush in a practical voltage range. In general, the coupling of the ionization and swelling of the brush when applying a surface potential offer another degree of freedom for tuning the charge storage.

**Fig. 12:**
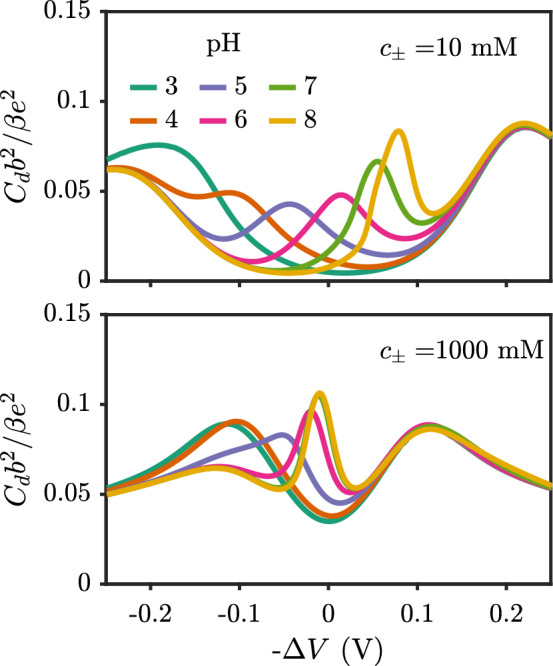
Differential capacitance for different values of pH with salt concentrations of (left) 10 mM and (right) 1000 mM. The chain length is $N=20$ and the grafting density is $\sigma _g b^2 = 0.01$

The capacitance has a nontrivial dependence on the pH and the salt concentration since both play a role in determining the equilibrium brush height. Figure 12 shows the varying behavior in the capacitance with pH and salt concentration. For each individual pH and salt concentration, the shape of the capacitance curve can be understood using the argument above, where the peak centered around zero potential is directly related to swelling of the brush. A benefit of the analysis above is that one can tune the capacitance by understanding the electroresponse of the brush height, rather than having to measure surface properties that may be difficult to access experimentally. In studies of ionic liquids, the shapes of the capacitance curves are often discussed [54]. The so-called *bird*-shaped capacitance curves appear qualitatively similar to those presented here. For ionic liquids, the bird-shaped capacitance results from the interplay between ion adsorption on an electrode and electrostatic repulsion of adsorbed species. Here, the prominent central peak is related to crossover from a more contracted to extended conformation due to the interplay of the osmotic pressure in the brush and the brush elasticity.

## Conclusion

For weak polyelectrolyte brushes, the interplay of the charge state, solution conditions, and electrostatic interaction with the surface leads to a variety of brush conformations. Recent experiments described an apparent paradox where applying a negative potential to a polyacid swells the brush, on the expectation that a negative potential should decrease the ionized fraction and contract the brush. This brush-centric view neglects the role of the surface charges. We rationalize the experimental observations through a scaling analysis that includes the surface charges and provide numerical results from an inhomogeneous thermodynamic theory that incorporates electrostatic correlations at the Debye–Hückel level. From the scaling analysis, the brush swelling mechanism is related to counterions compensating the surface charge density. We also compute the differential capacitance for weak polyelectrolyte brushes to highlight the connection between brush extension and charge storage in the brush.

Much is left to be explored for weak polyelectrolyte brushes, and we posit the brush swelling behavior can be used as a proxy to understand many surface properties, especially related to charge storage. In future studies, we hope to incorporate more molecular detail, such as nonelectrostatic interactions and a composition-dependent dielectric to better predict weak polyelectrolyte properties [39]. Open questions in this area includes elucidating the origin of hysteresis upon charging and discharging a surface with a weak polyelectrolyte brush [21], where nonelectrostatic effects likely play an important role in allowing a metastable collapsed or swollen state to persist; the role of polymer sequence in the electroresponse [55]; the effect of multivalent ions on weak polyelectrolyte brushes brush conformation since many of the brush scaling laws break down with multivalent ions for strong polyelectrolyte brushes [56–59]; and finally, the dynamics of brushes and small ions when applying an external stimuli, like an electric field. From a simulation and theory perspective, all of these questions require careful treatment of the coupling between the charge state and local solution conditions, which remains to be an outstanding challenge in the field.

### Supplementary Information

Below is the link to the electronic supplementary material.

Supplementary file 1 (pdf 232 KB)

## Data Availability

The datasets generated during and/or analyzed during the current study are available from the corresponding author on reasonable request.

## References

[1] Kreer T (2016). Polymer-brush lubrication: a review of recent theoretical advances. Soft Matter.

[2] Zhulina EB, Rubinstein M (2014). Lubrication by polyelectrolyte brushes. Macromolecules.

[3] Ali M, Yameen B, Neumann R, Ensinger W, Knoll W, Azzaroni O (2008). Biosensing and supramolecular bioconjugation in single conical polymer nanochannels. Facile incorporation of biorecognition elements into nanoconfined geometries. J. Am. Chem. Soc..

[4] Ma S, Ye Q, Pei X, Wang D, Zhou F (2015). Antifouling on Gecko’s feet inspired fibrillar surfaces: evolving from land to marine and from liquid repellency to algae resistance. Adv. Mater. Interfaces.

[5] Higaki Y, Kobayashi M, Murakami D, Takahara A (2016). Anti-fouling behavior of polymer brush immobilized surfaces. Polym. J..

[6] Borzȩcka NH, Kozłowska I, Gac JM, Bojarska M (2020). Anti-fouling properties of poly(acrylic acid) grafted ultrafiltration membranes - experimental and theoretical study. Appl. Surf. Sci..

[7] Pincus P (1991). Colloid stabilization with grafted polyelectrolytes. Macromolecules.

[8] Marins JA, Montagnon T, Ezzaier H, Hurel C, Sandre O, Baltrunas D, Mazeika K, Petrov A, Kuzhir P (2018). Colloidal stability of aqueous suspensions of polymer-coated iron oxide nanorods: implications for biomedical applications. ACS Appl. Nano Mater..

[9] Zhulina EB, Birshtein TM, Borisov OV (1995). Theory of ionizable polymer brushes. Macromolecules.

[10] Borukhov I, Andelman D, Borrega R, Cloitre M, Leibler L, Orland H (2000). Polyelectrolyte titration: theory and experiment. J. Phys. Chem. B.

[11] W. Kern, Der osmotische Druck wässeriger Lösungen polyvalenter Säuren und ihrer Salze: 215. Mitteilung über makromolekulare Verbindungen. Zeitschrift für Physikalische Chemie **184A**(1), 197–210 (1939). 10.1515/zpch-1939-18416

[12] Wang S, Granick S, Zhao J (2008). Charge on a weak polyelectrolyte. J. Chem. Phys..

[13] Nová L, Uhlík F, Košovan P (2017). Local pH and effective pKA of weak polyelectrolytes-insights from computer simulations. Phys. Chem. Chem. Phys..

[14] Zhulina EB, Rubinstein M (2012). Ionic strength dependence of polyelectrolyte brush thickness. Soft Matter.

[15] Nap RJ, Szleifer I (2018). Effect of calcium ions on the interactions between surfaces end-grafted with weak polyelectrolytes. J. Chem. Phys..

[16] Ferrand-Drake Del Castillo G, Hailes RL, Dahlin A (2020). Large changes in protonation of weak polyelectrolyte brushes with salt concentration-implications for protein immobilization. J. Phys. Chem. Lett..

[17] Lunkad R, Murmiliuk A, Tošner Z, Štěpánek M, Košovan P (2021). Role of pKA in charge regulation and conformation of various peptide sequences. Polymers.

[18] Gallegos A, Ong GMC, Wu J (2021). Thermodynamic non-ideality in charge regulation of weak polyelectrolytes. Soft Matter.

[19] Chen G, Das S (2016). Anomalous shrinking-swelling of nanoconfined end-charged polyelectrolyte brushes: interplay of confinement and electrostatic effects. J. Phys. Chem. B.

[20] Sachar HS, Pial TH, Desai PR, Etha SA, Wang Y, Chung PW, Das S (2020). Densely grafted polyelectrolyte brushes trigger “Water-in-Salt” like scenarios and ultraconfinement effect. Matter.

[21] Senechal V, Rodriguez-Hernandez J, Drummond C (2022). Electroresponsive weak polyelectrolyte brushes. Macromolecules.

[22] Nap R, Gong P, Szleifer I (2006). Weak polyelectrolytes tethered to surfaces: effect of geometry, acid-base equilibrium and electrical permittivity. J. Polym. Sci., Part B: Polym. Phys..

[23] Gallegos A, Ong GMC, Wu J (2021). Ising density functional theory for weak polyelectrolytes with strong coupling of ionization and intrachain correlations. J. Chem. Phys..

[24] Yamamoto T, Pincus PA (2011). Collapse of polyelectrolyte brushes in electric fields. Europhys. Lett..

[25] Ho YF, Shendruk TN, Slater GW, Hsiao PY (2013). Structure of polyelectrolyte brushes subject to normal electric fields. Langmuir.

[26] Longo GS, Olvera de la Cruz M, Szleifer I (2016). Controlling swelling/deswelling of stimuli-responsive hydrogel nanofilms in electric fields. Soft Matter.

[27] Okrugin BM, Richter RP, Leermakers FAM, Neelov IM, Zhulina EB, Borisov OV (2020). Electroresponsive polyelectrolyte brushes studied by self-consistent field theory. Polymers.

[28] Perez Sirkin YA, Szleifer I, Tagliazucchi M (2020). Voltage-triggered structural switching of polyelectrolyte-modified nanochannels. Macromolecules.

[29] Weir MP, Heriot SY, Martin SJ, Parnell AJ, Holt SA, Webster JRP, Jones RAL (2011). Voltage-induced swelling and deswelling of weak polybase brushes. Langmuir.

[30] Borisova OV, Billon L, Richter RP, Reimhult E, Borisov OV (2015). pH- and electro-responsive properties of Poly(acrylic acid) and Poly(acrylic acid)-block-poly(acrylic acid-grad-styrene) brushes studied by Quartz crystal microbalance with dissipation monitoring. Langmuir.

[31] Hollingsworth N, Larson RG (2021). Hysteretic swelling/deswelling of polyelectrolyte brushes and bilayer films in response to changes in pH and salt concentration. Polymers.

[32] Shen K, Wang Z-G (2017). Electrostatic correlations and the polyelectrolyte self energy. J. Chem. Phys..

[33] Ghasemi M, Larson RG (2021). Role of electrostatic interactions in charge regulation of weakly dissociating polyacids. Prog. Polym. Sci..

[34] Knoerdel AR, Blocher McTigue WC, Sing CE (2021). Transfer matrix model of pH effects in polymeric complex coacervation. J. Phys. Chem. B.

[35] Wu J (2006). Density functional theory for chemical engineering: from capillarity to soft materials. AIChE J..

[36] Jiang J, Cao D, Henderson D, Wu J (2014). Revisiting density functionals for the primitive model of electric double layers. J. Chem. Phys..

[37] Maribo-Mogensen B, Kontogeorgis GM, Thomsen K (2012). Comparison of the Debye-Hückel and the mean spherical approximation theories for electrolyte solutions. Ind. Eng. Chem. Res..

[38] Nakamura I, Wang Z-G (2012). Salt-doped block copolymers: ion distribution, domain spacing and effective parameter. Soft Matter.

[39] Nap RJ, Tagliazucchi M, Szleifer I (2014). Born energy, acid-base equilibrium, structure and interactions of end-grafted weak polyelectrolyte layers. J. Chem. Phys..

[40] Wertheim MS (1984). Fluids with highly directional attractive forces. II. Thermodynamic perturbation theory and integral equations. J. Stat. Phys..

[41] Wertheim MS (1987). Thermodynamic perturbation theory of polymerization. J. Chem. Phys..

[42] Zhang P, Wang Z-G (2021). Interfacial structure and tension of polyelectrolyte complex coacervates. Macromolecules.

[43] Koper GJ, Borkovec M (2010). Proton binding by linear, branched, and hyperbranched polyelectrolytes. Polymer.

[44] Petrov AI, Antipov AA, Sukhorukov GB (2003). Base-acid equilibria in polyelectrolyte systems: from weak polyelectrolytes to interpolyelectrolyte complexes and multilayered polyelectrolyte shells. Macromolecules.

[45] J. Choi, M.F. Rubner, Influence of the degree of ionization on weak polyelectrolyte multilayer assembly. Macromolecules **38**, 116–124 (2005). 10.1021/ma048596o

[46] Borisov OV, Zhulina EB, Birshtein TM (1994). Diagram of the states of a grafted polyelectrolyte layer. Macromolecules.

[47] Israëls R, Leermakers FA, Fleer GJ (1994). On the theory of grafted weak polyacids. Macromolecules.

[48] Ahrens H, Förster S, Helm CA (1998). Charged polymer brushes: counterion incorporation and scaling relations. Phys. Rev. Lett..

[49] O.V. Borisov, F.A.M. Leermakers, G.J. Fleer, E.B. Zhulina, Polyelectrolytes tethered to a similarly charged surface. J. Chem. Phys. **114**(17), 7700–7712 (2001). 10.1063/1.1360244

[50] Borisov OV, Boulakh AB, Zhulina EB (2003). Annealing polyelectrolytes at charged interfaces. The Eur. Phys. J. E.

[51] Seki H, Suzuki YY, Orland H (2007). Self-consistent field study of polyelectrolyte brushes. J. Phys. Soc. Jpn..

[52] Longo GS, Olvera De La Cruz M, Szleifer I (2014). Non-monotonic swelling of surface grafted hydrogels induced by pH and/or salt concentration. J. Chem. Phys..

[53] Tagliazucchi M, Calvo EJ, Szleifer I (2008). Redox and acid-base coupling in ultrathin polyelectrolyte films. Langmuir.

[54] Cruz C, Ciach A, Lomba E, Kondrat S (2019). Electrical double layers close to ionic liquid-solvent demixing. J. Phys. Chem. C.

[55] Li M, Zhuang B, Yu J (2021). Sequence-conformation relationship of Zwitterionic peptide brushes: theories and simulations. Macromolecules.

[56] Jiang T, Wu J (2008). Ionic effects in collapse of polyelectrolyte brushes. J. Phys. Chem. B.

[57] Yu J, Mao J, Yuan G, Satija S, Jiang Z, Chen W, Tirrell M (2016). Structure of polyelectrolyte brushes in the presence of multivalent counterions. Macromolecules.

[58] Yu J, Jackson NE, Xu X, Brettmann BK, Ruths M, de Pablo JJ, Tirrell M (2017). Multivalent ions induce lateral structural inhomogeneities in polyelectrolyte brushes. Sci. Adv..

[59] Li M, Zhuang B, Yu J (2022). Effects of Ion valency on polyelectrolyte brushes: a unified theory. Macromolecules.

