# Supplementary material for: Electroresponse of weak polyelectrolyte brushes
Source: Eur Phys J E Soft Matter. 2023 Sep 14;46(9):82. doi: 10.1140/epje/s10189-023-00341-3 (PMC10501941; doi:10.1140/epje/s10189-023-00341-3)
Supplement: Supplementary file 1 — (pdf 232 KB) [file 10189_2023_341_MOESM1_ESM.pdf]

Supplementary Information
Electroresponse of Weak Polyelectrolyte Brushes

Christopher Balzer [1] and Zhen-Gang Wang [1*]

[1]Division of Chemistry and Chemical Engineering, California Institute of Technology, 1200 E California Blvd, Pasadena, California 91125, USA.

*Corresponding author(s). E-mail(s): zgw@caltech.edu;

1 Potentials for Debye–Hückel Correlations

For clarity, we explicitly calculate the local chemical potential when all of the species are the same size

$$\frac{\delta \beta f_{\mathrm{DH}}}{\delta \rho_\alpha(\mathbf{r})} = -\frac{z_\alpha^2 \kappa(\mathbf{r}) l_B}{2(1+\kappa(\mathbf{r})d)} \tag{1}$$

$$\frac{\delta \beta f_{\mathrm{DH}}}{\delta \rho_w(\mathbf{x})} = -\frac{(s_w)^2 \kappa(\mathbf{r}) l_B}{2(1+\kappa(\mathbf{r})d)} \tag{2}$$

$$\frac{\delta \beta f_{\mathrm{DH}}}{\delta \rho_M(\mathbf{X})} = -\sum_{j=1}^{N} \frac{(s_j^M)^2 \kappa(\mathbf{r}) l_B}{2(1+\kappa(\mathbf{r})d)} \tag{3}$$

$$\frac{\delta \beta f_{\mathrm{DH}}^{\mathrm{ch}}}{\delta \rho_\alpha(\mathbf{r})} = -\sum_{j=1}^{N-1} \sum_{\{s_j^M\}} \sum_{\{s_{j+1}^M\}} \left[\rho_j^M(\mathbf{r}, s_j^M, s_{j+1}^M) + \rho_{j+1}^M(\mathbf{r}, s_j^M, s_{j+1}^M)\right] \frac{\zeta(\mathbf{r}, s_j^M, s_{j+1}^M)d}{(1+\kappa(\mathbf{r})d)} \frac{2\pi l_B z_\alpha^2}{\kappa(\mathbf{r})} \tag{4}$$

$$\frac{\delta \beta f_{\mathrm{DH}}^{\mathrm{ch}}}{\delta \rho_w(\mathbf{x})} = -\sum_{j=1}^{N-1} \sum_{\{s_j^M\}} \sum_{\{s_{j+1}^M\}} \left[\rho_j^M(\mathbf{r}, s_j^M, s_{j+1}^M) + \rho_{j+1}^M(\mathbf{r}, s_j^M, s_{j+1}^M)\right] \frac{\zeta(\mathbf{r}, s_j^M, s_{j+1}^M)d}{(1+\kappa(\mathbf{r})d)} \frac{2\pi l_B (s_w)^2}{\kappa(\mathbf{r})} \tag{5}$$

$$\frac{\delta\beta f^{\mathrm{ch}}_{\mathrm{DH}}}{\delta\rho_M(\mathbf{X})} = \sum_{j=1}^{N-1} \zeta(\mathbf{r}, s_j^M, s_{j+1}^M) - \rho_j^M(\mathbf{r}, s_j^M, s_{j+1}^M) \frac{\zeta(\mathbf{r}, s_j^M, s_{j+1}^M) d}{(1+\kappa(\mathbf{r})d)} \frac{2\pi l_B (s_j^M)^2}{\kappa(\mathbf{r})} + \sum_{j=1}^{N-1} \zeta(\mathbf{r}, s_j^M, s_{j+1}^M) - \rho_{j+1}^M(\mathbf{r}, s_j^M, s_{j+1}^M) \frac{\zeta(\mathbf{r}, s_j^M, s_{j+1}^M) d}{(1+\kappa(\mathbf{r})d)} \frac{2\pi l_B (s_{j+1}^M)^2}{\kappa(\mathbf{r})} \tag{6}$$

2 Effective Fields and Propagators for Weak Polymer

The effective field ω generally depends on the position $\mathbf{r}$ and monomer state s_j as well as adjacent states, s_{j-1} and s_{j+1}. For internal monomers,

$$\beta\omega(\mathbf{r}, s_{j-1}^M, s_j^M, s_{j+1}^M) = [\beta\epsilon_M + \beta\lambda - \beta e\psi(\mathbf{r})]\, s_j^M + \beta\eta(\mathbf{r}) + \frac{(s_j^M)^2 \kappa(\mathbf{r}) l_B}{2(1+\kappa(\mathbf{r})d)} - \zeta(\mathbf{r}, s_j^M, s_{j+1}^M) - \zeta(\mathbf{r}, s_{j-1}^M, s_j^M) + \frac{2\pi l_B d (s_j^M)^2}{(1+\kappa(\mathbf{r})d)\kappa(\mathbf{r})} \Big[\zeta(\mathbf{r}, s_j^M, s_{j+1}^M)\rho_j^M(\mathbf{r}, s_j^M, s_{j+1}^M) + \zeta(\mathbf{r}, s_{j-1}^M, s_j^M)\rho_j^M(\mathbf{r}, s_{j-1}^M, s_j^M)\Big] \tag{7}$$

For the chain ends,

$$\beta\omega(\mathbf{r}, s_1^M, s_2^M) = [\beta\epsilon_M + \beta\lambda - \beta e\psi(\mathbf{r})]\, s_1^M + \beta\eta(\mathbf{r}) + \frac{(s_1^M)^2 \kappa(\mathbf{r}) l_B}{2(1+\kappa(\mathbf{r})d)} + \zeta(\mathbf{r}, s_1^M, s_2^M) \left[\frac{2\pi l_B d (s_1^M)^2 \rho_1^M(\mathbf{r}, s_1^M, s_2^M)}{(1+\kappa(\mathbf{r})d)\kappa(\mathbf{r})} - 1\right] \tag{8}$$

and

$$\beta\omega(\mathbf{r}, s_{N-1}^M, s_N^M) = [\beta\epsilon_M + \beta\lambda - \beta e\psi(\mathbf{r})]\, s_N^M + \beta\eta(\mathbf{r}) + \frac{(s_N^M)^2 \kappa(\mathbf{r}) l_B}{2(1+\kappa(\mathbf{r})d)} + \zeta(\mathbf{r}, s_{N-1}^M, s_N^M) \left[\frac{2\pi l_B d (s_N^M)^2 \rho_N^M(\mathbf{r}, s_{N-1}^M, s_N^M)}{(1+\kappa(\mathbf{r})d)\kappa(\mathbf{r})} - 1\right] \tag{9}$$

The chain propagators can be defined recursively from the initial conditions of $q_1(\mathbf{r}, s_1^M, s_2^M) = e^{\beta\omega(\mathbf{r}, s_1^M, s_2^M)}\delta(\mathbf{r} - \mathbf{r}_\perp)$ and $q_N^*(\mathbf{r}, s_{N-1}^M s_N^M) = e^{\beta\omega(\mathbf{r}, s_{N-1}^M, s_N^M)}$, where the $\delta(\mathbf{r})$ is the Dirac delta function that is used to constrain the grafted chain end to the surface. For $j = 2$,

$$q_2(\mathbf{r}, s_1^M, s_2^M, s_3^M) = e^{\beta\omega(\mathbf{r}, s_1^M, s_2^M, s_3^M)} \int d\mathbf{r}' \Phi(|\mathbf{r} - \mathbf{r}'|) q_1(\mathbf{r}', s_1^M, s_2^M) \tag{10}$$

For $j \in [3, N-1]$,

$$q_j(\mathbf{r}, s_{j-1}^M, s_j^M, s_{j+1}^M) = e^{\beta\omega(\mathbf{r}, s_{j-1}^M, s_j^M, s_{j+1}^M)} \int d\mathbf{r}' \Phi(|\mathbf{r}-\mathbf{r}'|) \sum_{s_{j-2}^M} q_{j-1}(\mathbf{r}', s_{j-2}^M, s_{j-1}^M, s_j^M) \tag{11}$$

For $j = N$,

$$q_N(\mathbf{r}, s_{N-1}^M, s_N^M) = e^{\beta\omega(\mathbf{r}, s_{N-1}^M, s_N^M)} \int d\mathbf{r}' \Phi(|\mathbf{r}-\mathbf{r}'|) \sum_{s_{N-2}^M} q_{N-1}(\mathbf{r}', s_{N-2}^M, s_{N-1}^M, s_N^M) \tag{12}$$

For the second to last monomer ($j = N-1$), the complimentary propagator is

$$q_{N-1}^*(\mathbf{r}, s_{N-2}^M, s_{N-1}^M, s_N^M) = e^{\beta\omega(\mathbf{r}, s_{N-2}^M, s_{N-1}^M, s_N^M)} \int d\mathbf{r}' \Phi(|\mathbf{r}-\mathbf{r}'|) q^*(N, \mathbf{r}', s_{N-1}^M, s_N^M) \tag{13}$$

For $j \in [2, N-2]$, the complimentary propagator is

$$q_j^*(\mathbf{r}, s_{j-1}^M, s_j^M, s_{j+1}^M) = e^{\beta\omega(\mathbf{r}, s_{j-1}^M, s_j^M, s_{j+1}^M)} \int d\mathbf{r}' \Phi(|\mathbf{r}-\mathbf{r}'|) \sum_{s_{j+2}^M} q_{j+1}^*(\mathbf{r}', s_j^M, s_{j+1}^M, s_{j+2}^M) \tag{14}$$

For $j = 1$, the complimentary propagator is

$$q_1^*(\mathbf{r}, s_1^M, s_2^M) = e^{\beta\omega(\mathbf{r}, s_1^M, s_2^M)} \int d\mathbf{r}' \Phi(|\mathbf{r}-\mathbf{r}'|) \sum_{s_3^M} q_1^*(\mathbf{r}', s_1^M, s_2^M, s_3^M) \tag{15}$$

3 Free Energy and Potential for Chi Interactions

For the polymer species M, the χ potential can be written as

$$F_\chi = -\chi \int d\mathbf{r}' \rho_M(\mathbf{r}')^2 \tag{16}$$

Taking the variation with respect to the total density

$$\frac{\delta F_\chi}{\delta \rho_M(\mathbf{X})} = -2\chi \sum_{j=1}^{N} \rho_M(\mathbf{r}) \tag{17}$$

4 Additional Figures

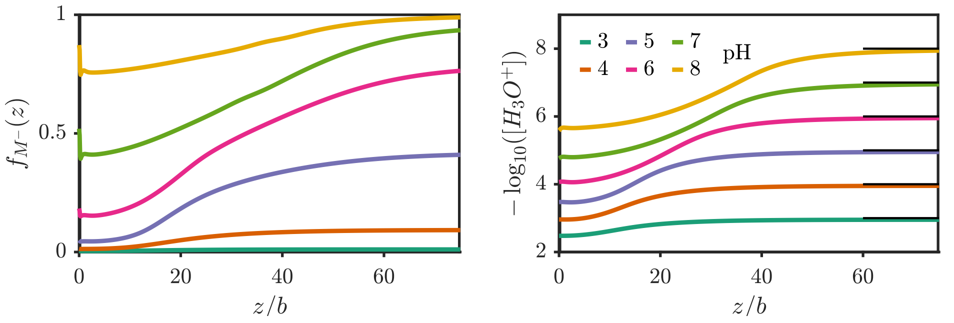

Fig. S1 Ionized fraction and hydronium concentration near a neutral surface for different values of pH with $pK_{a,0} = 5$. (left) Local ionization fraction of polyacid brush. (right) Local concentration of hydronium. The salt concentration is 10 mM, the chain length is $N = 50$ and the grafting density is $\sigma_g b^2 = 0.03$.

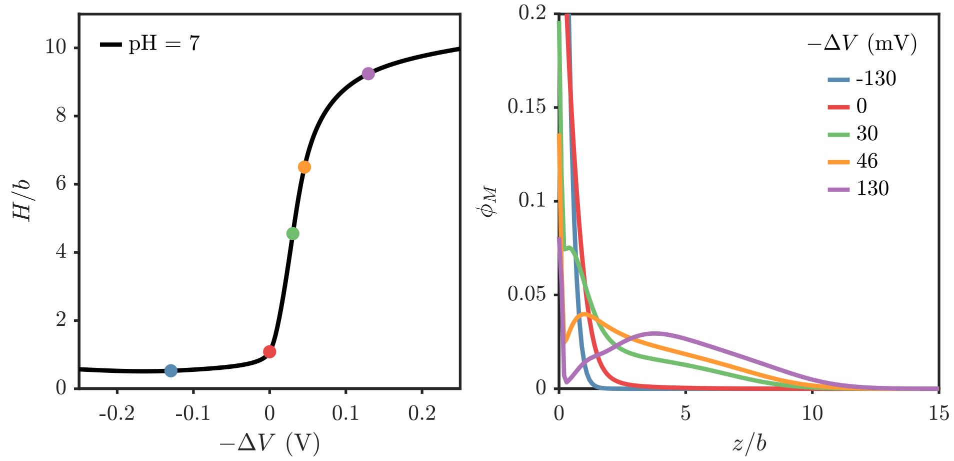

Fig. S2 Effect of applied potential on the brush conformation and brush profiles for $pK_{a,0} = 5$ at pH=7. (left) Brush height as a function of potential and (right) density profiles for selected potentials with colors corresponding to the circles in the left figure. The salt concentration is 100 mM, the chain length is $N = 20$, and the grafting density is $\sigma_g b^2 = 0.01$.
